# Ferroptosis in non-alcoholic liver disease: Molecular mechanisms and therapeutic implications

**DOI:** 10.3389/fnut.2023.1090338

**Published:** 2023-03-13

**Authors:** Zilu Cheng, Huikuan Chu, Qingjing Zhu, Ling Yang

**Affiliations:** ^1^Division of Gastroenterology, Union Hospital, Tongji Medical College, Huazhong University of Science and Technology, Wuhan, Hubei, China; ^2^Jinyintan Hospital, Huazhong University of Science and Technology, Wuhan, China

**Keywords:** ferroptosis, iron metabolism, nonalcoholic liver disease, time-restricted feeding, enoyl coenzyme A hydratase 1

## Abstract

Ferroptosis refers to a novel modality of regulated cell death characterized by excessive iron accumulation and overwhelming lipid peroxidation, which takes an important part in multiple pathological processes associated with cell death. Considering the crucial roles of the liver in iron and lipid metabolism and its predisposition to oxidative insults, more and more studies have been conducted to explore the relationship between ferroptosis and various liver disorders, including non-alcoholic fatty liver disease (NAFLD). With increased morbidity and high mortality rates, NAFLD has currently emerged as a global public health issue. However, the etiology of NAFLD is not fully understood. In recent years, an accumulating body of evidence have suggested that ferroptosis plays a pivotal role in the pathogenesis of NAFLD, but the precise mechanisms underlying how ferroptosis affects NAFLD still remain obscure. Here, we summarize the molecular mechanisms of ferroptosis and its complicated regulation systems, delineate the different effects that ferroptosis exerts in different stages of NAFLD, and discuss some potential effective therapies targeting ferroptosis for NAFLD treatment, which putatively points out a novel direction for NAFLD treatment.

## 1. Introduction

Cell death is widely identified as a pivotal partaker in mammalian development, homeostasis regulation, and a variety of diseases, which also has an intimate association with other physiological processes (1). Cell death can be roughly grouped in two categories: regulated cell death and accidental cell death. Ferroptosis, as an ancient modality of regulated cell death, is extensively observed in mammals (2). Moreover, a kind of ferroptosis-like death also seems to exert its function in plants, protozoa, and fungi (3–5). Dixon et al. (2) firstly put forward the term of ferroptosis in 2012, which refers to a form of cell death driven by overwhelming lipid peroxidation in an iron-dependent manner (1). Ferroptosis exhibits distinct genetic, morphological, and biochemical features compared to other modalities of cell death, including apoptosis, autophagy as well as necrosis (2, 6, 7). Under electron microscope, the typical morphological characteristics of ferroptosis consist of the paucity of nuclear chromatin agglutination, mitochondrial cristae reduction, mitochondrial atrophy, and cellular membrane rupture (8). Moreover, ferroptosis is often concomitant with lipid reactive oxygen species (ROS) accumulation, glutathione (GSH) depletion, glutathione peroxidase 4 (GPX4) inhibition, and the alterations of the gene profile related to the regulation of iron metabolism and lipid peroxidation (8). Evidence is mounting that ferroptosis plays an important role in various disorders, including ischemia/reperfusion, cancer, and neurodegenerative diseases (9–11). Recently, considering the pivotal roles of the liver in iron and lipid metabolism and its predisposition to oxidative insults, more and more studies have been conducted to explore the relationship between ferroptosis and liver disorders, including non-alcoholic fatty liver disease (NAFLD) (12–15).

Non-alcoholic fatty liver disease is a chronic metabolic liver disease without other secondary causes or excessive alcohol consumption, which consists of a constellation of liver disorders, including simple steatosis and non-alcoholic steatohepatitis (NASH) (16). The incidence of NAFLD is increasing year by year, and currently, the individuals suffering from NAFLD account for 25% of the world’s population (17). If not given timely intervention, the patients with NASH will further advance to cirrhosis and hepatocellular carcinoma (HCC) (18). Moreover, NAFLD is also intimately related to chronic kidney diseases (19), cardiovascular diseases (20), and extrahepatic cancer (21), which greatly threatens human health. The etiology of NAFLD is multifactorial, and steatosis, insulin resistance (IR), iron overload, oxidative stress, and mitochondrial dysfunction are generally deemed to be great contributors to the initiation and progression of NAFLD (22–24). With more intensive investigations of ferroptosis, a plethora of studies have suggested that ferroptosis is able to induce oxidative stress, exacerbate inflammatory responses, and cause cell damage, which putatively expedites the pathological process of NAFLD (25–27).

Considering the prevalence of NAFLD and its detrimental effects on human health, it is urgent to seek effective therapeutic methods for the disease. Lifestyle interventions including proper dietary regimen and moderate exercise can exert marked effect in the early stage of NAFLD, but when the disease progresses to the advanced stages, like fibrosis or cirrhosis, simple lifestyle interventions are unable to achieve satisfactory results, which highly demands the emergence of effective pharmacological agents (28). Given that many researches have indicated that there is an intimate relationship between ferroptosis and NAFLD (26, 27), it is highly necessary to unravel the precise mechanisms underlying how ferroptosis affects NAFLD, and discuss the efficacy and safety of several potential therapeutic interventions targeting ferroptosis for NAFLD treatment.

## 2. Ferroptosis

### 2.1. Iron metabolism

#### 2.1.1. Iron absorption and transport

Iron, as an indispensable trace element, plays a critical role in various biological processes in mammals, including cellular signaling transaction, DNA replication, mitochondrial respiration and oxygen delivery (29). However, iron also can be harmful owing to its unique function in inducing ROS production, which can exert marked influence on the normal structure and function of proteins, nucleic acids, and lipids (30). Herein, both iron deficiency and iron overload greatly threaten human health. The main sources of iron in the human body include food and aging erythrocytes. Among which, dietary iron can be further grouped into heme iron and non-heme iron. The heme iron is mainly present in the blood and red meat of animals, and is readily absorbed by the human gut (31). In contrast, the non-heme iron is extensively distributed in the plant food and is difficult to be absorbed (31). The non-heme iron from the plant food is mainly composed of Fe^3+^, and its absorption can be influenced by multiple dietary factors. Specifically, Vitamin C, animal tissues and several proteins from meat potentiate non-heme iron absorption in the gut while calcium, phytate, polyphenol, and some proteins from eggs inhibit it (31). The duodenal cytochrome b (DCb) and divalent metal-iron transporter 1 (DMT1) distributed in duodenum are in charge of reducing non-heme Fe^3+^ to Fe^2+^ and absorbing Fe^2+^ in the gut lumen, respectively (32, 33). The heme iron from the animal food is primarily made up of Fe^2+^, which is scarcely affected by dietary factors on top of calcium (31). Calcium has the capacity to reduce the absorption of heme iron and non-heme iron, but the specific mechanisms by which calcium inhibits iron uptake has not been thoroughly figured out (34). The heme iron is similarly absorbed by duodenal enterocytes, but the specific molecules and mechanisms underlying how heme iron is absorbed by the duodenum still remain obscure. The absorbed heme iron in enterocytes is further degraded by heme oxygenase (HO), and then Fe^2+^ is released, which can be exported to the circulation through the transport proteins hephaestin and ferroportin 1 located on the basolateral membrane of enterocytes along with the absorbed non-heme iron (35–37).

The Fe^2+^ in circulation absorbed from the gut, together with that released from aging erythrocytes, is mainly oxidized to Fe^3+^ with the assistance of ceruloplasmin. Subsequently, the Fe^3+^ combines to transferrin and forms a complex, which then is transported to various tissues and organs throughout the body (7, 38). The transferrin receptor 1 (Trf1) located on cells is able to mediate the endocytosis of the complex composed of Fe^3+^ and transferrin, resulting in the import of iron into cells (38). In addition, a part of iron can be directly imported into cells by SLC39A14 in the form of Fe^2+^, further being released to labile iron pool (LIP) (39). LIP refers to an unstable and transitory pool of ferrous iron within the cells, which is redox-reactive and chelatable, serving as an important intersection of metabolic pathways of iron-contained substances. Intracellular Fe^3+^ can be reduced to Fe^2+^ in the lysosome by six transmembrane epithelial antigens of prostate 3, and subsequently, zinc-iron regulatory protein family 8/14 or DMT1 leads to the release of Fe^2+^ from the lysosome into LIP (40, 41). Moreover, a part of Fe^3+^ in cells can also be stored in ferritin (42). Nuclear receptor coactivator 4 has the capacity to recognize ferritin and mediate ferritinophagy, which refers to a selective modality of autophagy and is responsible for the autophagic degradation of ferritin, ultimately reducing stored Fe^3+^ to Fe^2+^ and releasing them into LIP (42–44). Furthermore, ferroportin is responsible for the export of Fe^2+^ from cells, which is selectively expressed in the cells related to the transfer of iron to circulation, such as periportal hepatocytes, splenic red pulp macrophages, hepatic Kupffer cells as well as duodenal enterocytes (45). The exported Fe^2+^ is firstly oxidized to Fe^3+^ with the assistance of hephaestin, and then, Fe^3+^ combines to the transferrin in circulation, which can be utilized by other cells (36).

#### 2.1.2. Regulation of iron metabolism

##### 2.1.2.1. Several critical regulatory factors

Under physiological conditions, iron metabolism is sophisticatedly regulated, and the dynamic equilibrium of iron is well maintained. Hepcidin, as a hepatic hormone, plays a pivotal role in the regulation of iron metabolism, which predominantly takes ferroportin as target (46). Hepcidin has the capacity to induce the endocytosis and degradation of ferroportin and block its open-outward conformation, thereby inhibiting the release of iron into systematic circulation (47, 48). The inflammatory cytokines, like interleukin-6, hepatic iron store, and plasma iron concentration are all recognized as significant regulators of hepcidin (49, 50). The hypoxia-inducible factor (HIF)-2α regulated by iron and oxygen can modulate intestinal iron absorption by directly targeting several critical intestinal iron transporters, including ferroportin, DMT1 and DCb (51). The nuclear erythroid-related factor 2 (Nrf2), as one of significant regulators in antioxidant responses, is able to induce the transcription of ferritin heavy chain and HO1 (52). The upregulation of HO1 is highly related to the increment of Fe^2+^ in LIP, as HO1 is able to promote the release of Fe^2+^ in heme (53). As an intracellular iron chaperone, PCBP1 has the capacity to transport iron to the proteins requiring no-heme iron (54). And the specific knockout of PCBP1 in hepatocytes was confirmed to increase LIP in mice (55).

##### 2.1.2.2. Gut microbiota

The gut microbiota colonizes in the human digestive tract and exerts extensive impact on multiple physiological and pathological processes of the host. Several studies have found that compared to the conventional counterparts, the levels of stored iron were reduced in the kidney, spleen and liver of germ-free mice and rabbits (46, 56). And the body iron retention in germ-free rats could be increased by 25% after conventionalizing them (57). These results potently linked the gut microbiota to iron metabolism. Iron is not only indispensable for multiple biological processes in mammals but also essential for bacterial survival (58). The iron fortifiers absorbed in the duodenum just account for 5% to 15% of the total, while the rest is utilized by resident microorganisms in the colon (59). The gut microbiota putatively modulates iron metabolism *via* their intrinsic enzymes associated with iron uptake and transport (60), the synthesis of siderophore, as well as the production of several microbial metabolites that are viewed as potent regulators in iron metabolism (46). To be more specific, bacterial cytochrome b561 is able to affect intestinal iron absorption by competing with the DCb distributed on enterocytes (60). Moreover, a substantial body of gut bacteria can release iron-chelating siderophores to acquire intestinal iron (61). Compared to the iron-binding protein of the host, the siderophore exhibits higher affinity to iron (61). In a recent study, two microbial metabolites, reuterin and 1,3-diaminopropane produced by *Lactobacillus reuteri* and *Escherichia coli*, respectively, could inhibit iron accumulation in the iron overload model mice by repressing HIF-2α (46), which is crucial for the modulation of intestinal iron absorption as mentioned previously. Several acidic metabolites produced by gut bacteria, like short chain fatty acids and lactic acids, can effectively lower gut luminal pH, which is conducive to reducing Fe^3+^ within the gut lumen to more absorbable Fe^2+^ (62, 63).

In summary, the dietary iron is predominantly absorbed in the duodenum *via* DCb and DMT1, and then, ferroportin 1 and hephaestin mediates the translocation of iron from enterocytes to systematic circulation (32, 33, 35, 36). Most of free iron absorbed from the gut and released from aging erythrocytes firstly binds to transferrin (7, 38). Subsequently, the iron-ferritin complex is imported into cells *via* Trf1 (38). Besides, a part of iron can be directly imported into cells in the form of Fe^2+^ (39). After entering into cells, the iron can be released into LIP or stored in ferritin (40–42). Furthermore, in some cells related to the transfer of iron to plasma, the iron within cells can also be exported to circulation with the assistance of ferroportin (45). At a healthy state, the dynamic equilibrium of iron is well maintained and the iron metabolism is regulated by a variety of factors, including hepcidin (47, 48), HIF-2α (51), Nrf2 (52), and the gut microbiota (46). If the iron metabolism is dysregulated and the LIP is enlarged, the overloaded iron, especially Fe^2+^, initiates the Fenton reaction to produce a large quantity of ROS, which can further trigger lipid peroxidation and subsequent ferroptosis (64).

### 2.2. Lipid peroxidation

**FIGURE 1:**
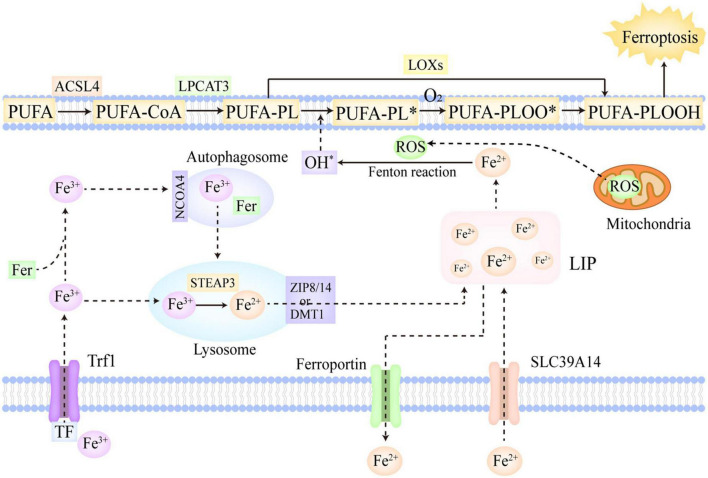
The molecular mechanisms of ferroptosis. Fe^3+^ is the predominant form of iron in circulation, which often combines with Trf and can be imported into cells *via* Trf1 located on cellular membrane. In addition, a part of iron can be imported into cells by SLC39A14 in the form of Fe^2+^. The Fe^3+^ within cells is firstly reduced to Fe^2+^ in the lysosome catalyzed by STEAP3, and then, ZIP 8/14 or DMT1 leads to the release of Fe^2+^ from the lysosome into LIP. Moreover, the Fe^3+^ in cells can also be stored in ferritin. NCOA4 can recognize ferritin and mediate ferritinophagy, reducing stored Fe^3+^ to Fe^2+^ and ultimately releasing Fe^2+^ into LIP. Furthermore, ferroportin is mainly in charge of the export of Fe^2+^ from cells. If the iron metabolism is disrupted, excessive Fe^2+^ will trigger lipid peroxidation and ferroptosis. PUFAs, as the most predominant substrates of lipid peroxidation, are firstly converted to PUFA-PLs by ACSL4 and LPCAT3. In the enzymatic pathway, LOXs oxidize PUFA-PLs to PUFA-PLOOH. In the non-enzymatic pathway, the excessive Fe^2+^ firstly reacts with ROS to generate OH∙ *via* Fenton reaction, which then abstracts hydrogen from PUFA-PL and forms PUFA-PL∙. Subsequently, O_2_ oxidizes PUFA-PL∙ to PUFA-PLOO∙, which abstracts hydrogen from the adjacent PUFA-PL and facilitates the propagation of lipid peroxidation. Ultimately, the overwhelming lipid peroxidation results in cellular membrane destruction and ferroptosis. ACSL4, acyl-CoA synthetase long-chain family member 4; NCOA4, nuclear receptor coactivator 4; LIP, labile iron pool; DMT1, divalent metal-iron transporter 1; LOXs, lipoxygenases; STEAP3, six transmembrane epithelial antigens of prostate 3; Trf, transferrin; Trf1, transferrin receptor 1; SLC39A14, solute carrier family 39 member 14; ZIP 8/14, zinc-iron regulatory protein family 8/14; PUFAs, polyunsaturated fatty acids; ROS, reactive oxygen species; LPCAT3, lysophosphatidylcholine acyltransferase 3. The symbol * means free radical.

Lipids are identified as one of the most important nutrients needed by the human body, which not only provide the body with needed energy and essential fatty acids, but also constitute indispensable components of human cells and tissues. Lipid peroxidation is referred to the oxidative degeneration of lipids, which can seriously damage the lipids-containing molecules and structures, such as lipoprotein and cellular membranes (65). Lipid peroxidation disrupts the integrity of cellular membranes by altering their fluidity and permeability, thereby exerting marked influence on cellular function (66). A substantial body of studies have verified that lipid peroxidation participates in the development of various disorders, including atherosclerosis, ischemia/reperfusion, and neurodegenerative diseases (67–69). In pace with more intensive investigations of cell death, lipid peroxidation is currently regarded as a hallmark of ferroptosis (65). Collectively, lipid peroxidation is initiated by two classic pathways, including the enzymatic pathway and non-enzymatic pathway (Figure 1).

Polyunsaturated fatty acids (PUFAs) are generally deemed to be the most predominant substrates of lipid peroxidation. Notably, it is esterified PUFAs (phospholipids of polyunsaturated fatty acids, PUFA-PLs), not free PUFAs, that can induce ferroptosis after they are oxidized (70). In the PUFA-PLs biosynthesis, acyl-CoA synthetase long-chain family member 4 (ACSL4) is in charge of acetylizing PUFAs, and then, lysophosphatidylcholine acyltransferase 3 (LPCAT3) catalyzes the insertion of PUFAs-CoA into lysophospholipids, ultimately forming PUFA-PLs (71). The lipoxygenase (LOX) family is mainly made up of a constellation of non-heme iron-containing enzymes, and in humans, it includes LOX5, LOX12, LOX12B, LOX15, LOX15B, and LOXE3, which greatly contributes to the production of various lipid hydroperoxides in the enzymatic pathway (72). Arachidonic acid (AA), as a kind of PUFAs, is crucial in the induction of ferroptosis (7). AA is firstly transformed to AA-PL by ACSL4 and LPCAT3, and subsequently, AA-PL is oxidized to AA-phospholipid hydroperoxide (PLOOH) directly by LOX12 and LOX15, and indirectly by LOX5 which requires cytosolic phospholipase A2 to hydrolyze the esterified AA (66). Ultimately, the formed AA-PLOOH leads to the destruction of cell membrane and induce ferroptosis (73). In the non-enzymatic pathway, the free liable iron (Fe^2+^) and ROS occupy the core position in lipid peroxidation. Hydrogen peroxide and superoxide radical anions are the main intracellular ROS, which are predominantly generated in mitochondria. Berson et al. (74) The cellular excessive Fe^2+^ can react with these ROS and further convert them into OH∙ *via* the Fenton reaction (75). OH∙ has the capacity to abstract hydrogen from PUFA-PL, thus forming PUFA-PL∙, which can be further oxidized to PUFA-PLOO∙ by O_2_ (70). The formed PUFA-PLOO∙ is able to abstract hydrogen from the adjacent PUFA-PL, generating PUFA-PLOOH and a new PUFA-PL∙. Then, the new PUFA-PL∙ is oxidized to PUFA-PLOO∙ by O_2_ and reacts with the next PUFA-PL, thus facilitating the further propagation of lipid peroxidation (70). Besides the two aforementioned classic pathways, cytochrome P450 oxidoreductase (POR) was also found to make contributions to the initiation of lipid peroxidation in recent studies (76). POR provided electrons for CYB5A and cytochrome P450 through using NAPDH as electron donors. Then, the reduced CYB5A and cytochrome P450 could reduce Fe^3+^ to Fe^2+^, and abstract methylene hydrogen from PUFAs, thus indirectly and directly triggering lipid peroxidation (76, 77).

In conclusion, PUFAs, as the most predominant substrates of lipid peroxidation, are firstly converted to PUFA-PLs by ACSL4 and LPCAT3 (71). In the enzymatic pathway, LOXs oxidize PUFA-PLs to PUFA-PLOOH (72). In the non-enzymatic pathway, the excessive Fe^2+^ firstly reacts with ROS to generate OH∙ *via* the Fenton reaction (75), which then leads to the abstraction of hydrogen from PUFA-PL and the production of PUFA-PL∙ (70). Subsequently, O_2_ oxidizes PUFA-PL∙ to PUFA-PLOO∙, which abstracts hydrogen from the adjacent PUFA-PL and facilitates the propagation of lipid peroxidation (70). Furthermore, in recent studies, POR was also found to make contributions to the initiation of lipid peroxidation. The overwhelming lipid peroxidation results in the destruction of cellular membrane and ferroptosis (73).

### 2.3. Major mechanisms implicated in ferroptosis regulation

#### 2.3.1. System Xc^–^/GSH/GPX4 axis

Glutathione peroxidase 4, as one kind of selenoprotein, is widely deemed to be the major enzyme conferring protection against cellular membrane damage induced by lipid peroxidation, as it can reduce toxic lipid peroxides to non-toxic alcohols (78). Yang et al. (79) have found that the genetic knockout of GPX4 in mice was intimately related to rapid lipid peroxides accumulation, which ultimately induced ferroptosis. Consistent with this, it has also been observed that the reduced cell viability caused by lipid ROS could be reversed by the ectopic expression of GPX4 (80). These results addressed the significance of GPX4 in lipid peroxidation. The two electrons predominantly provided by GSH and the selenocysteine residue of GPX4 in charge of catalyzing are essential in the process of reducing toxic lipid peroxides to non-toxic alcohols (81). GSH is the most abundant low molecular polypeptide in mammals, which is composed of glutamate, cysteine, and glycine. It not only participates in iron-sulfur synthesis, but also acts as an important reductant cofactor for multiple enzymes, including GPX4 (82). The system Xc^–^ refers to a cystine/glutamate reverse transporter that is located on cellular membrane, which encompasses two subunits, including solute carrier family 7 member 11 (SLC7A11) and SLC3A2 (83). SLC7A11 exhibits a high specificity to glutamate and cystine, and is mainly in charge of the transmembrane transport of the two amino acids, whereas SLC3A2 acts as a chaperonin, which has an intimate association with the transport of SLC7A11 to cellular membrane (83). The system Xc^–^ simultaneously transfers glutamate extracellularly and cystine intracellularly at a 1:1 ratio (2, 84). The cystine imported into cells can be further reduced to cysteine, which is widely deemed to be a rate-limiting substrate for GSH synthesis (85). Taken together, the system Xc^–^ contributes to the production of GSH by transferring cystine intracellularly, and GSH is a crucial cofactor for GPX4, which can reduce toxic lipid peroxides to non-toxic alcohols. Herein, the system Xc^–^/GSH/GPX4 axis in the cytosol can be regarded as a potent regulator in lipid peroxidation, and the inhibition of this axis putatively accelerates the pathological process of ferroptosis (86). Of note, mitochondrial GPX4 has also been identified as another significant cellular protective mechanism against ferroptosis, as it greatly contributed to the detoxification of mitochondrial lipid peroxides, reducing mitochondrial ferroptosis (87).

#### 2.3.2. FSP1/CoQ axis

In pace with more intensive investigations of ferroptosis, several studies have indicated that ferroptosis suppressor protein 1 (FSP1) also played a significant role in ferroptosis independent of the system Xc^–^/GSH/GPX4 axis (88, 89). Bersuker et al. (88) have found that FSP1 had the capacity to mediate ferroptosis resistance in the mouse tumor xenograft, and that the expression of FSP1 was positively related to the resistance to ferroptosis in hundreds of cancer cell lines. Additionally, Doll et al. (89) have observed a reduction of lipid peroxidation products in the FSP1 overexpressing cell whose GPX4 was knocked out previously, and the increased sensitivity to ferroptosis in FSP1-knockout cells. FSP1 was known as the flavoprotein apoptosis-inducing factor mitochondria-associated 2 (AIFM2) previously, which exhibited pro-apoptotic properties (90). The specific function of FSP1/AIFM2 depends on its cellular location. The translocation of FSP1 from mitochondria to cellular membrane leads to the switch of its pro-apoptotic properties to anti-ferroptotic properties, which mainly relies on the myristoylation of FSP1 (91). The FSP1 located on cellular membrane can reduce ubiquinone (CoQ10) to ubiquinol, which has the capacity to terminate lipid peroxidation by directly reducing lipid radicals (88). Moreover, FSP1 also can promote the regeneration of vitamin E, which is identified as one of the most potent antioxidants in the human body and protects cells against free radicals (92). Furthermore, independent of cytosol FSP1, dihydroorotate dehydrogenase (DHODH) was found to inhibit mitochondrial ferroptosis by reducing CoQ10 to ubiquinol, which represents a novel protective system against ferroptosis (87).

#### 2.3.3. GCH1/BH4 axis

The GTP cyclohydrolase-1 (GCH1)/Tetrahydrobiopterin (BH4) axis was deemed to be another independent pathway in the regulation of ferroptosis in several recent studies which performed the genome-wide screen (93, 94). The overexpression of GCH1 was confirmed to confer potent protection against ferroptosis induced by GPX depletion, IKE, and RSL3 (94). GCH1 exerts its anti-ferroptotic effect mainly by GCH1/BH4 axis. GCH1 catalyzes the rate-limiting step of BH4 synthesis, thus markedly affecting BH4 concentration in the body (95). BH4 not only takes part in numerous central metabolic processes by serving as an important cofactor for various enzymes (96), but also downregulates the oxidative degradation of phospholipids contained two PUFA tails by serving as a direct antioxidant and an important regulator in CoQ_10_ synthesis (94), thus inhibiting lipid peroxidation and inducing ferroptosis.

#### 2.3.4. The associated transcriptional factors

P53 is generally regarded as a classic tumor suppressor, which plays a pivotal role in modulating multiple biological processes, including energy metabolism, cell cycle, senescence, and apoptosis (95). Evidence was accumulating that P53 putatively exerted its anti-tumor effects *via* the induction of ferroptosis (97). It was able to trigger ferroptosis by downregulating SLC7A11, which is an important constituent of the system Xc^–^ as mentioned previously (97). However, P53 was also reported to repress ferroptosis by combining with dipeptidyl peptidase-4, which could interact with NOX1 on cellular membrane and lead to lipid peroxidation without P53 (98).

As a critical transcription factor, Nrf2 participates in various biological processes, such as proteostasis, iron metabolism, antioxidant responses and so on (99). Recent studies have also identified the significant regulatory role of Nrf2 in ferroptosis. Under basal conditions, Nrf2 undergoes continuous ubiquitination and degradation, and combines with cytoplasm kelch like epichlorohydrin-related protein-1 (Keap1), thus keeping a low concentration in the body (100). Oxidative stress is thought to induce the alteration of Keap1 conformation and the subsequent release of Nrf2 from Keap1. The released Nrf2 is then translocated into the nucleus in which it promotes the transcription of multiple genes containing antioxidant response element (ARE) (100), thus markedly alleviating oxidative insults. Moreover, Nrf2 can also act as a significant upstream modulator of numerous critical genes involved in ferroptotic cascades, like GPX4 and HO1 (100). Furthermore, Nrf2 participates in the regulation of metabolism and thus correlates with the production of some important metabolites in the redox system, including GSH and NAPDH (95). Therefore, it may be safe to draw a conclusion that Nrf2 has the capacity to regulate lipid peroxidation and ferroptosis *via* multiple mechanisms.

Besides the aforementioned classic P53 and Nrf2, there are many other transcription factors participating in ferroptosis regulation in different conditions, including aryl hydrocarbon receptor nuclear translocator like (ARNTL) in circadian rhythm disorder, activating transcription factor (ATF) in endoplasmic reticulum stress, hypoxia inducible factor (HIF) in hypoxia as well as heat shock transcription factors (HSF) in hyperthermia (98).

**FIGURE 2:**
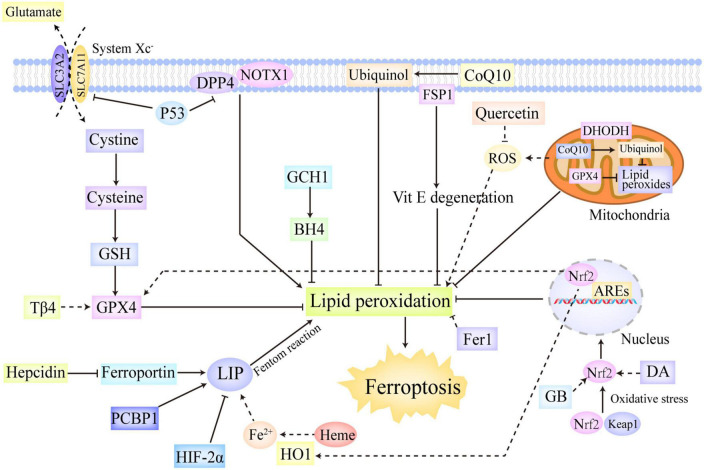
The regulation systems involved in ferroptosis. Ferroptotic cascades are under sophisticated and multifaceted regulation. The system Xc^–^/GSH/GPX4 axis reduces toxic lipid peroxides to non-toxic alcohols, thus markedly downregulating lipid peroxidation. The FSP1/CoQ axis can reduce CoQ10 to ubiquinol and regenerate Vitamin E, which confer protection against free radical insults and lipid toxicity. The GCH1/BH4 axis not only exerts direct antioxidant effects but also regulates the synthesis of CoQ10, thereby inhibiting lipid peroxidation and ferroptosis. In addition, mitochondrial GPX4 and DHODH were also found to represent two additional defensive enzymes against ferroptosis *via* the detoxification of lipid peroxides in the mitochondria. Moreover, P53 and Nrf2 are identified as two significant transcriptional factors in ferroptosis regulation. P53 not only can trigger ferroptosis by downregulating SLC7A11, but also can repress ferroptosis by combining with DPP4, which interacts with NOX1 on cellular membrane and leads to lipid peroxidation without P53. Oxidative stress initiates the release of Nrf2 from Keap1, thus activating Nrf2. The activated Nrf2 is then translocated into the nucleus in which it can regulate the transcription of the genes contained AREs, thus conferring protection against oxidative insults. Besides, Nrf2 also participates the regulation of some important genes in ferroptotic cascades, such as HO1 and GPX4. Furthermore, hepcidin, HIF-2α, HO1, and PCBP1 also take part in ferroptosis regulation by exerting impact on iron metabolism. ARE, antioxidant response element; BH4, tetrahydrobiopterin; CoQ10, ubiquinone; DHODH, dihydroorotate dehydrogenase; DPP4, dipeptidyl peptidase-4; GCH1, GTP cyclohydrolase-1; FSP1, ferroptosis suppressor protein 1; GSH, glutathione; GPX4, glutathione peroxidase 4; HIF, hypoxia-inducible factor; HO1, heme oxygenase 1; Keap1, kelch like epichlorohydrin-related protein-1; SLC7A11, solute carrier family 7 member 11; Nrf2, nuclear erythroid-related factor 2.

In summary, ferroptotic cascades are under sophisticated and multifaceted regulation (Figure 2). The system Xc^–^/GSH/GPX4 axis is able to reduce toxic lipid peroxides to non-toxic alcohols, thus markedly downregulating lipid peroxidation (78). The FSP1/CoQ axis can reduce CoQ10 to ubiquinol and regenerate Vitamin E, which confers protection against free radical insults and lipid toxicity (88, 89, 92). The GCH1/BH4 axis not only exerts direct antioxidant effects but also regulates the synthesis of CoQ10, thereby inhibiting lipid peroxidation and ferroptosis (94). Of note, mitochondrial GPX4 and DHODH also represented two additional defensive enzymes against ferroptosis *via* the detoxification of lipid peroxides in the mitochondria (87). Moreover, there are a plethora of critical transcription factors involved in ferroptosis regulation, including P53, Nrf2, ARNTL, ATF, HIF, and HSF (97, 98, 100). Furthermore, as mentioned previously, hepcidin, HIF-2α, HO1, and PCBP1 also participate in ferroptosis regulation by exerting impact on iron metabolism (51–55).

## 3. The role of ferroptosis in NAFLD

Iron overload and lipid peroxidation are generally identified as main characteristics of ferroptosis. The liver takes an important part in iron metabolism, including iron absorption, utilization, storage, and secretion (55). The liver is also deemed to be the most significant iron reservoir which is responsible for the storage of about one-third of total iron in the human body (101). The dysregulation of iron metabolism in the liver putatively brings the production of tremendous ROS, which markedly increases the susceptibility of hepatocytes to ferroptosis (101). Additionally, the disequilibrium of iron homeostasis was confirmed to correlate with IR and obesity (102). Taken together, it appears rational to infer that ferroptosis participates in the pathogenesis of NAFLD, as iron overload is an important contributor to NAFLD. A plethora of studies have provided massive compelling evidence to support this notion (22, 103). The elevated serum ferritin level was often observed in the patients with NAFLD, and it tended to be concomitant with higher NAFLD activity score, contributing to the identification of patients with advanced NAFLD (22). Yang et al. (103) found that dietary iron intake had a positive correlation with the prevalence of NAFLD in a dose-responsive way. And iron overload induced by metabolic disorders was reported to exacerbate liver injury in patients with NASH (104, 105). Aberrant iron accumulation in the liver putatively resulted from the downregulation of iron-export protein ferroportin 1 and the upregulation of iron-import proteins DMT1 and Trf1 in NAFLD, which enhanced intestinal iron absorption and promoted the bioavailability of iron (106–108). As mentioned above, excessive iron is able to initiate the Fenton reaction, which contributes to the production of lipid ROS and the execution of ferroptosis. Ferroptosis has been found to show a positive correlation with hepatic steatosis and inflammation in NAFLD. By establishing a NASH mouse model exposed to methionine/choline-deficient (MCD) diet, Qi et al. (25) found that the condition of liver steatosis and inflammation in these model mice was markedly aggravated by RSL3 (a ferroptosis inducer) treatment. In contrast, it was markedly improved by liproxstatin 1 and deferoxamine mesylate salt (ferroptosis inhibitors) treatment (25). Similarly, in another animal study, the administration of DFP and Trolox (ferroptosis inhibitors) in NASH model mice exposure to choline-deficient, ethionine-supplemented diet, reduced the peroxidation of phosphatidylethanolamine, alleviated hepatic inflammatory responses, and inhibited necrotic cell death in the liver (109). Furthermore, the depletion of iron by phlebotomy therapy was also confirmed to significantly improve liver histology of NAFLD patients in a phase 2 trial (110). These studies potently linked ferroptosis to the pathogenesis of NAFLD.

### 3.1. Aggravating hepatic steatosis

Hepatic steatosis is a fundamental feature of NAFLD, and abnormal lipid accumulation in hepatocytes acts as a critical driver of the development of NAFLD. A recent bio-informatics analysis has suggested that there existed an intimate relationship between the grade of hepatic steatosis and the expression of several genes involved in ferroptosis, such as GSS, ACSL4, ACSL3 and so on (111). Enoyl coenzyme A hydratase 1 (ECH1) treatment in the MCD-induced NASH mice was confirmed to mitigate hepatic steatosis and liver damage by blocking ferroptosis (112). The putative mechanisms by which ECH1 blocked ferroptosis included the inhibition of the extracellular signal regulated kinase (Erk) pathway and the upregulation of GPX4 (112). In another animal study, ferroptosis inducer RSL3 aggravated lipid accumulation in the liver of NASH model mice induced by MCD, which was abrogated by an iron-chelating agent, DFO (25). In line with this, lipid accumulation and the levels of hepatic triglycerides in MCD-fed mice were markedly reduced after treated with ferroptosis inhibitor ferrostatin-1 (Fer1) (26). As indicated above, the increased LIP in ferroptosis contributes to the formation of highly reactive ROS (such as OH∙) *via* the Fenton reaction, which then reacts with PUFAs-PLs contained in cellular membrane, leading to the depletion of long chain PUFAs. Long chain PUFAs, such as ω-3 long chain PUFAs, play important roles in regulating lipid metabolism in the liver, which not only represses hepatic lipogenesis by inhibiting the activation of SREBP1c, but also promotes fatty acids β oxidation by activating PPARα (113). In addition, long chain n-3 PUFAs supplementation showed great protection against hepatic steatosis and IR in NAFLD (114). Thus, it appears plausible to speculate that the depletion of long chain PUFAs in ferroptosis putatively contributes to hepatic steatosis in NAFLD. Moreover, oxidative stress caused by ferroptosis also exacerbates hepatic steatosis by triggering unfolded protein responses, which has the capacity to upregulate hepatic lipogenesis through its two branches (113). Collectively, the above referred evidence supported the notion that inducing ferroptosis greatly contributed to hepatic steatosis in NASH/NAFLD by facilitating hepatic lipogenesis and abnormal lipid accumulation in the liver.

### 3.2. Eliciting inflammatory responses

Chronic inflammation is identified as one of primary features of NAFLD, which serves as a pathological critical point between non-alcoholic fatty liver and NASH. Hepatic inflammation not only directly causes liver damage, but also contributes to the pathological progression of IR and liver fibrosis. Inflammatory cytokines can interfere IR/IRS/PI3K signaling pathway by activating a series of kinases, such as IKKβ, thus causing IR (115). Moreover, inflammatory mediators can also act as a potent activator of hepatic stellate cells (HSCs), which results in extracellular matrix deposition and liver fibrosis (116). As aforementioned, ferroptosis inducers exacerbated inflammatory responses in NASH model mice, whereas ferroptosis inhibitors alleviated inflammatory injury in such subjects (25, 104, 105, 117). These evidences indicated that ferroptosis putatively aggravated hepatic inflammation in the progression of NAFLD. It is commonly accepted that ferroptosis, as a modality of cell death, is usually concomitant with compromised cellular membrane and the release of cellular constituents, which can recruit a number of immune cells, further eliciting strong inflammatory responses (8). Of note, the released free iron from ferroptotic hepatocytes greatly contributes to the initiation of inflammatory cascades. The macrophages in the liver can recycle massive free iron released from ferroptotic hepatocytes (118). In turn, iron accumulation in macrophages facilitates its polarization to proinflammatory types (118). Iron has also been reported to participate in the regulation of tricarboxylic acid cycle in macrophages, which further modulated the production of inflammatory cytokines (118). Moreover, the regulation of inflammatory areas by liver resident macrophages could be disturbed by iron, which resulted in the recruitment of massive immune cells to the damaged areas and the release of proinflammatory cytokines from immune cells into circulation (118). Furthermore, iron overload can promote the production of ROS in T cells, which leads to DNA damage and undermines the responsive ability of T cells, thus expediting the pathological process of inflammation (119). Several studies have also noted that ferroptosis was intimately related to higher PTGS2 (a gene coding COX2) expression and AA metabolism (79). The COX2 catalyzes the production of prostaglandin, which acts as a significant inflammatory mediator. Additionally, in a high-fat diet (HFD)-induced NAFLD rat model, the alterations in AA concentration were identified as an early hallmark of inflammation and had a positive correlation with the severity of inflammation (120). However, in a phase 2 study, the inflammation condition was not significantly improved by the depletion of iron (110). More efforts are still warranted to explicitly identify the role that ferroptosis plays in inflammatory cascades of NAFLD patients.

### 3.3. Exerting a dual effect in liver fibrosis

Liver fibrosis refers to an intricate pathological process in the liver characterized by excessive accumulation of extracellular matrix (121). It has an intimate association with chronic liver damage and acts as a significant intermediate pathogenetic link of multiple chronic liver disorders, such as NAFLD (122). If not treated timely, liver fibrosis putatively leads to more detrimental events, including cirrhosis, liver failure, HCC, and even death. Recently, many researchers have studied the relationship between ferroptosis and liver fibrosis, but the results they attained were controversial. When administrated with a high-iron diet, the transferrin-knockout mice were susceptible to develop ferroptosis-induced liver fibrosis, which was significantly ameliorated with Fer1 treatment (12). The results supported the notion that ferroptosis exacerbated liver fibrosis. However, there still were many other studies indicating that the induction of ferroptosis in HSCs could markedly alleviated liver fibrosis, as the activation of HSCs was viewed as an indispensable pathological factor for liver fibrosis. In an animal study, artesunate exerted anti-fibrosis properties by inducing ferritinophagy-mediated HSC ferroptosis (13). The animal experiments performed in model mice with liver fibrosis delineated that artemether treatment significantly mitigated liver fibrosis by inducing ferroptosis in HSCs in a P53-dependent way (14). Similarly, magnesium isoglycyrrhizinate, a safe product from natural glycyrrhizic acids, was also confirmed to ameliorate liver fibrosis *via* the induction of ferroptosis in HSCs, which was mainly attributed to the upregulation of HO1 (15). The controversial effects of ferroptosis in liver fibrosis are putatively attributed to the contrary functions of ferroptosis in hepatocytes and HSCs. Specifically, in the development of liver fibrosis, hepatocyte ferroptosis exacerbates liver injury, whereas HSC ferroptosis mitigates liver injury (53). Herein, it is critical to conduct more intensive investigations to clarify the definite relationship between ferroptosis and liver fibrosis, and to develop a drug delivery system which can target the specific cell types in the liver.

## 4. Potential effective therapies targeting ferroptosis for NAFLD

### 4.1. Ferroptosis inhibitors

Fer1 is a selective and potent ferroptosis inhibitor, which is separated through high-throughput screening of the small molecule library (123). Owing to its ability to scavenge alkoxyl radicals generated by Fe^2+^ from lipid hydroperoxides, Fer1 exerts anti-ferroptosis effects (123). In a NASH mouse model induced by MCD diet, Fer1 treatment was confirmed to reverse hepatic inflammation and fibrosis induced by MCD diet through blocking ferroptosis *via* the neutralization of lipid ROS (26).

As one kind of multifunctional polypeptides, thymosin beta 4 (Tβ4) is extensively distributed in various nucleated cells. In previous studies, Tβ4 was found to reduce the levels of ROS (124), and the contents of serum Tβ4 in NAFLD patients were decreased significantly (125). Recently, in order to identify the role of Tβ4 in NAFLD, some researches have established a NAFLD mouse model induced by HFD, and found that Tβ4 upregulated the expression of GPX4, and further reduced oxidative stress and lipid peroxidation, ultimately inhibiting ferroptotic cascades and ameliorating the inflammatory damage of the liver (126).

Quercetin is widely accepted as an important flavonoid, which exerts anti-inflammatory and anti-oxidant effects, and is abundant in teas, herbs, fruits and vegetables (127). A plethora of studies have confirmed that quercetin treatment was conducive to the alleviation of HFD-induced NAFLD *via* the restoration of gut microbiota and the improvement of lipid metabolism and inflammation condition (128, 129). Additionally, in a recent study, quercetin was also found to confer protection against NAFLD by inhibiting ferroptosis in the liver, which had an intimate association with the reduction of mitochondrial ROS in hepatocytes (130).

Ginkgolide B (GB), as a predominant constituent of Ginkgo biloba extracts, takes an important part in neuroprotective, anti-inflammatory, and anti-apoptotic activities (131, 132). In the HFD-induced NAFLD model mice, GB markedly ameliorated oxidative injury and lipid peroxidation by blocking ferroptosis (133). The mechanism by which GB inhibited ferroptosis showed a strong correlation with Nrf2 activation, which was able to regulate the expression of several ferroptosis-related proteins, including FTH1, TFR1, HO1 and GPX4, thus promoting iron metabolism and repressing lipid peroxidation (133).

Dehydroabietic acid (DA), a natural tricyclic diterpenoid resin acid separated from coniferous plants, exhibits potent anti-inflammatory, antisenescence, anti-bacterial, and anti-tumor properties (134–137). In a recent animal study, DA was confirmed to have the capacity to bind to Keap1, which resulted in the release of Nrf2 from Keap1, thus activating Nrf2 (138). The activated Nrf2 could reduce lipid peroxidation and ferroptosis by regulating the transcription of some critical genes involved in ferroptosis (GSH, GSS, GST, and HO1), which ultimately improved HFD-induced NAFLD in model mice (138). Moreover, the levels of FSP1 and CoQ10 were also increased after DA treatment, which could potently block ferroptosis as mentioned above (138).

### 4.2. Time-restricted feeding

Time-restricted feeding (TRF) is defined as regular calorie intake at the indicated time, and consuming food during other time is rigorously banned (139). It is of note that TRF does not influence the total calorie intake. In some animal researches, TRF was confirmed to improve lipid metabolism disorder, glucose tolerance, as well as hepatic steatosis and injury (140). In order to explore the relationship between TRF and NASH and its associated mechanisms, Shu et al. (139) have established a NASH mouse model induced by high-fat and high-fructose diet. They found that ferroptosis was induced in such mice, and that TRF significantly ameliorated NASH by inhibiting ferroptosis *via* the downregulation of Per2 gene’s expression (139). Per2, as a circadian gene, plays a significant role in the regulation of circadian rhythm and glucose and lipid metabolism, and the paucity of Per2 gene and the dysfunction of Per2 protein led to the reduction of serum glucose and triacylglycerol levels, as well as body weight (141, 142). In Shu et al.’s (139) study, Per2 was confirmed to promote ferroptosis, as the knockout of Per2 in hepatocytes showed a strong correlation with ferroptosis inhibition. Moreover, TRF, hepatocyte Per2 knockout, and ferroptosis inhibition all promoted the expression of PPARα, an important partaker in the pathogenesis of NASH, suggesting that PPARα was likely to be the downstream molecule by which ferroptosis regulated NASH (139). Herein, TRF represents a new therapeutic option for NAFLD/NASH.

### 4.3. Enoyl coenzyme A hydratase 1

Enoyl coenzyme A hydratase 1, as the key enzyme in the second step of mitochondrial fatty acid β-oxidation, has the capacity to regulate a variety of pathophysiological processes, including conferring protection against metabolic disorders and obesity (143). Previously, ECH1 has been confirmed to mitigate HFD-induced IR and hepatic steatosis directly by restraining the expression of lipogenesis genes and by repressing the insulin pathway *via* the attenuation of Akt phosphorylation (144). In a recent animal study, some researchers have found that ECH1 also showed an intimate correlation with NASH. ECH1 overexpression was able to ameliorate hepatic steatosis, fibrosis, and liver damage in the MCD-induced NASH model mice *via* the inhibition of ferroptosis (112). In contrast, ECH1 knockdown expedited the pathological progression of NASH, which could be reversed by ferroptosis inhibitor Fer1 (112). Moreover, in this study, the putative mechanisms by which ECH1 blocked ferroptosis were described as the inhibition of the Erk pathway and the upregulation of GPX4 (112). Among which, inhibiting Erk pathway has been confirmed to show protection against ferroptosis in C2C12 and C57810 cells (145). But notably, the specific cell type in the liver which ECH1 targets still remain unknown, which awaits more intensive study.

In summary, ferroptosis inhibitors, TRF, and ECH1 show great potential in the treatment of NAFLD (Table 1). Ferroptosis inhibitors, such as Fer1 (26), Tβ4 (126), Quercetin (130), GB (133), and DA (138), had the capacity to reduce oxidative stress and lipid peroxidation, inhibit inflammatory responses, and ameliorate liver damage in the animal models of NASH/NAFLD. TRF was able to improve NASH by inhibiting ferroptosis *via* the downregulation of circadian Per2 gene’s expression (139). ECH1 also ameliorated hepatic steatosis and fibrosis, and liver damage in NASH model mice by repressing ferroptosis *via* the inhibition of the Erk pathway and the upregulation of GPX4 (112). Furthermore, besides the aforementioned methods directly targeting ferroptosis and associated pathways, restraining iron uptake also represents a putative therapeutic approach for NAFLD, as the activity of iron uptake in the gut was much higher in NAFLD/NASH patients (146). In a case-control study, Peng et al. (147) found that reducing the consumption of animal-derived iron showed a strong correlation with reduced NAFLD risk. In addition, an increased level of the iron-import protein DMT1 was observed in NAFLD subjects, and some DMT1 inhibitors have been designed (146). However, in the treatment of NAFLD, these inhibitors could not exert satisfactory effects (148). Therefore, great efforts are warranted to develop more effective inhibitors targeting iron-import proteins and enhancers targeting iron-export proteins, which can reduce intestinal iron absorption and block ferroptosis, thus conferring protection against NAFLD.

**TABLE 1:** Potential effective therapies targeting ferroptosis for NAFLD.

Therapeutic interventions	Ferroptosis	Target	Mechanisms	Therapeutic implications
Ferrostatin 1 (26)	Inhibitor	Lipid ROS	Inhibits lipid peroxidation	Reverses serum ALT and AST elevation in NASH; downregulates the expression of proinflammatory and fibrogenesis genes
Thymosin beta 4 (126)	Inhibitor	GPX4	Inhibits lipid peroxidation	Ameliorates hepatic inflammatory damage in NAFLD
Quercetin (130)	Inhibitor	Mitochondrial ROS	Inhibits oxidative stress	Improves the pathological condition of NAFLD
Ginkgolide B (133)	Inhibitor	Nrf2	Promotes the transcription of genes containing ARE, GPX4, etc.	Improves the pathological condition of NAFLD
Dehydroabietic acid (138)	Inhibitor	Nrf2	Promotes the transcription of genes containing ARE, GPX4, etc.	Improves the pathological condition of NAFLD
TRF (139)		Per 2	Downregulates the expression of Per 2	Improves the pathological condition of NASH
ECH1 (112)		GPX4	Promotes the expression of GPX4	Improves the pathological condition of NASH

NAFLD, non-alcoholic fatty liver disease; ALT, alanine aminotransferase; AST, aspartate aminotransferase; NASH, non-alcoholic steatohepatitis; GPX4, glutathione peroxidase 4; ROS, reactive oxygen species; Nrf2, nuclear erythroid-related factor 2; ARE, antioxidant response element; TRF, time-restricted feeding; ECH1, enoyl coenzyme A hydratase 1.

## 5. Conclusion

Ferroptosis is referred to a modality of regulated cell death driven by overwhelming lipid peroxidation in an iron-dependent manner (1). It is intimately related to lipid toxicity, ROS accumulation, insulin resistance, and inflammatory insults, which are regarded as great contributors to the initiation and development of NAFLD (22–24). A substantial body of animal and human studies have provided compelling evidence to support the notion that ferroptosis takes an important part in NAFLD pathogenesis (25, 103, 117). Interestingly, ferroptosis exerts different effects in different stages of the disease. Specifically, ferroptosis contributes to hepatic lipogenesis and abnormal lipid accumulation in the liver, and expedites the development of simple steatosis to NASH by eliciting inflammatory responses in the liver (8, 118, 119). When NAFLD progresses to liver fibrosis, ferroptosis seems to have dual effects (12–15). On the one hand, it exacerbates liver damage by disrupting normal hepatocytes (53). On the other, it ameliorates liver fibrosis by inactivating HSCs (53). Furthermore, ferroptosis inhibitors, TRF, and ECH1 which take ferroptosis as target have been confirmed to have great potential in the treatment of NAFLD in many animal studies (26, 126, 130, 149, 150).

However, there still exists many unsolved issues concerning ferroptosis in NAFLD. Firstly, most of researches exploring the role of ferroptosis in NAFLD were conducted in animal models, which does not necessarily apply to humans. Secondly, the key cell type for ferroptosis activation in the NAFLD process has not been figured out. Thirdly, which lipids plays the central role in ferroptosis of NAFLD and the related downstream pathways by which these lipids induce ferroptosis still remain obscure. Fourthly, although it is obvious that the gut microbiota pattern in NAFLD is different from that in healthy individuals, and that gut microbiota participates in the regulation of ferroptosis, whether the gut microbiota contributes to the development of NAFLD by modulating ferroptosis and which the specific strains produce a marked effect in this process still are unanswered questions. Fifthly, the role of ferroptosis in liver fibrosis is controversial, which needs more intensive investigation. Sixthly, most of potential therapeutic interventions targeting ferroptosis for NAFLD are only utilized in animal studies, not in clinical practice, and ferroptosis inducers not only attack pathogenic cells, but also damage normal liver cells. It is highly necessary to develop useful drug delivery systems which can target the specific cell types in the liver. Addressing these important questions left unanswered is conducive to better understanding the specific role of ferroptosis in NAFLD, and providing solid theoretical foundation for the formation of more effective and precise treatment guidelines for NAFLD.

## Author contributions

ZC and HC collected the literatures and drafted the manuscript. LY and QZ contributed to the conception and design of the work and critically revised the manuscript. All authors read and approved the final version of the manuscript.
